# Mechanical Characterization at the Microscale of Mineralized Bone Callus after Bone Lengthening

**DOI:** 10.3390/ma15186207

**Published:** 2022-09-07

**Authors:** Flavy Roseren, Sandrine Roffino, Martine Pithioux

**Affiliations:** 1 Aix Marseille Univ, CNRS, ISM, 13009 Marseille, France; 2 Aix Marseille Univ, APHM, CNRS, ISM, Sainte-Marguerite Hospital, Institute for Locomotion, Department of Orthopaedics and Traumatology, 13009 Marseille, France; 3 Aix Marseille Univ, APHM, CNRS, ISM, Mecabio Platform, Anatomy Laboratory, 13009 Marseille, France

**Keywords:** distraction osteogenesis, microindentation, mineralized callus, bone regeneration, mechanical properties

## Abstract

Distraction osteogenesis (DO) involves several processes to form an organized distracted callus. While bone regeneration during DO has been widely described, no study has yet focused on the evolution profile of mechanical properties of mineralized tissues in the distracted callus. The aim of this study was therefore to measure the elastic modulus and hardness of calcified cartilage and trabecular and cortical bone within the distracted callus during the consolidation phase. We used a microindentation assay to measure the mechanical properties of periosteal and endosteal calluses; each was subdivided into two regions. Histological sections were used to localize the tissues. The results revealed that the mechanical properties of calcified cartilage did not evolve over time. However, trabecular bone showed temporal variation. For elastic modulus, in three out of four regions, a similar evolution profile was observed with an increase and decrease over time. Concerning hardness, this evolves differently depending on the location in the distracted callus. We also observed spatial changes in between regions. A first duality was apparent between regions close to the native cortices and the central area, while latter differences were seen between periosteal and endosteal calluses. Data showed a heterogeneity of mechanical properties in the distracted callus with a specific mineralization profile.

## 1. Introduction

Distraction osteogenesis (DO) is a surgical technique that allows correction of leg length discrepancy and short stature whose etiology could be multifactorial, such as congenital disease, infection, or post-traumatic injury [1]. The process of bone regeneration during DO is long and complex. It involves multiple cellular (proliferation, migration, differentiation…) and physiological processes (angiogenesis, bone regeneration process, and mecanotransduction) that lead to the formation and maturation of a distracted callus [2,3,4,5]. The latter is composed of several tissues whose properties change over time. In the latency phase, formation of a hematoma and granulation tissue are apparent and form the soft callus [3]. Then, during the distraction phase, gradual and controlled traction of osteotomized bone segments results in the formation of an immature woven bone and then trabecular bone. Finally, once the desired elongation has been achieved, the consolidation phase begins. When the distraction forces stop, the fixator is held in place until the distracted callus is fully grown and mineralized [6].

The particularity of bone formation during DO is that there is a spatial and temporal organization of tissues during the bone regeneration process. In the literature, the bone regenerating process during DO has been widely described through analyses of histological, radiological, molecular, and cellular changes [2,7,8,9,10,11,12]. Even though some characterizations of mechanical properties of the bone callus have already been established at the macroscopic scale thanks to three-point bending or tensile tests [11,13,14,15], no study has yet focused on the evolution of the elastic modulus and the hardness of the distracted callus at a microstructural level. The use of indentation as a research tool to analyze the bone microstructure is not widespread. There are only three studies that have used an indentation assay to characterize the mechanical properties of bone callus at microscale. Two studies were done in a bone-healing process [16,17] and one during bone transport in the metatarsal in a sheep model [18]. It is important to quantify the mechanical properties of the tissues present in the bone callus to determine the heterogeneity during the mineralization of bone-forming tissues, which will influence the mechanical strength of the structure at a tissue level [19,20]. This will make it possible to link the progressive recovery of the mechanical function of the bone tissue to the underlying biological processes of bone regeneration. In addition, the major interest of deepening knowledge on bone quality during regeneration could make it possible, on the one hand, to promote biomedical engineering, and on the other hand, to enrich the numerical models of bone mechanobiology.

In this context, the goal of this study was to assess the elastic modulus and hardness of the mineralized tissues within the distracted callus (spatial characterization) at different time points in the consolidation phase (temporal characterization). In order to obtain local variations of the elastic modulus and hardness, we needed to identify the different tissues inside the callus (calcified cartilage, trabecular bone, and cortical bone). To do this, the indentation study was performed on thick serial sections following the antero-posterior axis of the callus, and tissues were characterized by histological study to guide the indentation. Using this methodology, we obtained the mechanical evolution profile of the bone regeneration process over time according to a specific region in the distracted callus and the spatial variation of mechanical properties inside the distracted callus by comparing different regions of interest (periosteal callus and endosteal callus).

## 2. Materials and Methods

### 2.1. Sample Preparation

**Figure 1:**
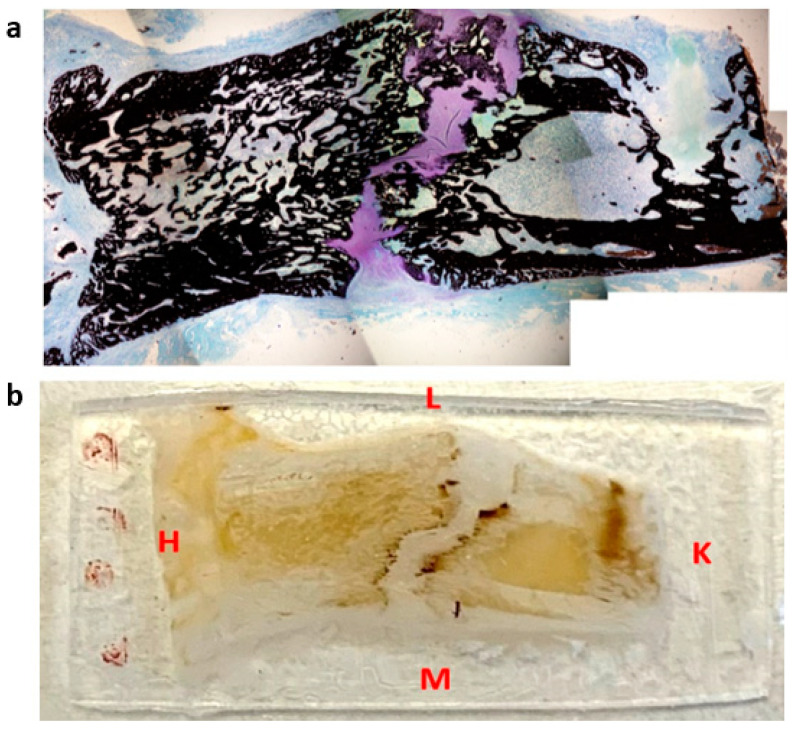
Representation of histological section (**a**) and microindentation (**b**) of a distracted callus harvested at 45-DPS. L = lateral, M = medial, H = hip, K = knee. The sections were cut following the antero-posterior axis in the frontal plane.

Animal experiments were conducted in accordance with the European directives 2010/63. All procedures described in this project were approved by the institutional animal care and use committee of the University of Aix-Marseille and by the French Ministry of Research. Animals were housed in individual cages with 12 h light/dark cycles and temperature-controlled rooms. Rats were closely monitored during the entire experiment and were fed a standard laboratory diet ad libitum. Male Sprague–Dawley rats (500 g ± 20) were assigned for a surgery of callotasis on the right femur according to an established protocol [21]. Briefly, DO consists of a latency period of 7 days, followed by a 10-day distraction phase where the external fixator is activated at the rate of 0.5 mm/day with one incrementation of 0.25 mm every 12 h, leading to a total elongation of 5 mm. At the end of the distraction phase, the consolidation phase started. Distracted calluses were harvested at different time points during the consolidation period: two at 31-days post-surgery (31-DPS), two at 45-days post-surgery (45-DPS), and one at 59-days post-surgery (59-DPS)—since one animal died due to a broken pin leading to a misalignment of the bone segments. Prior to the resin embedding, the distracted callus samples were resized with a dental saw under constant hydration to isolate the callus and the adjacent cortical bone. Then, the samples were prepared for Methyl methacrylate resin embedding. Following that, the embedded samples were mounted on a microtome (Leica RM 2265, Wetzlar, Germany) and trimmed in the longitudinal plane with a D-profile tungsten knife until we could observe the four native cortices on the same section. When reached, a 7 µm thick section was cut for histological staining (Figure 1a). Immediately after this, the embedded samples were mounted on a water-cooled, low-speed diamond saw (Buehler Isomet 4000, Buehler, Lake Bluff, IL, USA) and a 400 µm thick section was cut for indentation assay as shown in Figure 1b. Once harvested, the embedded samples were mounted back on the microtome and these steps were repeated until the disappearance of the four native cortices. For each distracted callus, two to three indentation sections could be made, depending on the callus morphology.

### 2.2. Histological Staining

**Figure 2:**
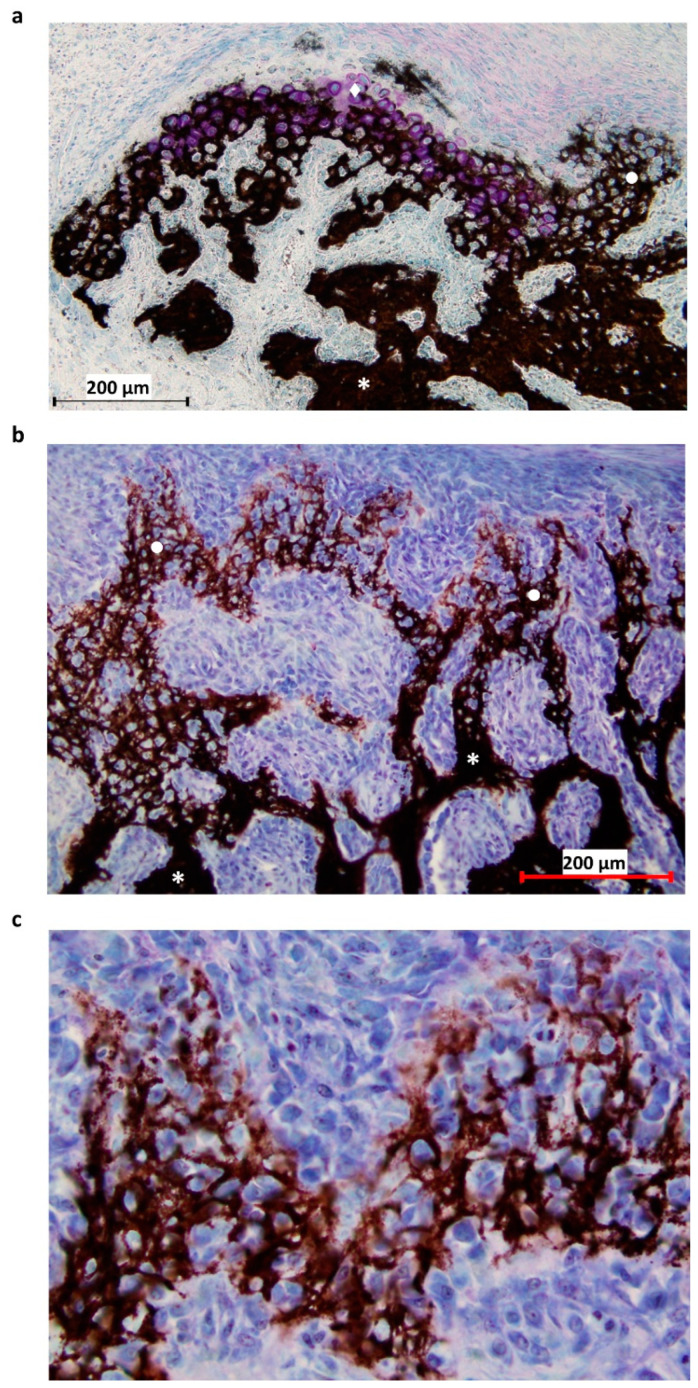
Histological section of the P1 area at 31-DPS. (**a**) Identification of mineralized tissue by using Von Kossa staining. On the top left, newly-calcified cartilage is formed as the chondrocytes (pink coloration) are still present. On the right, calcified cartilage has already been deposited and can be recognized by its honeycomb formation. (**b**) Other area in the P1 region, where the transition between calcified cartilage and trabecular bone can be seen. Trabecular bone is present at the bottom of the image and calcified cartilage at the top. (**c**) Magnification ×20 of calcified cartilage present in the distracted callus. * = trabecular bone, ● = calcified cartilage, ◆ = calcified cartilage in formation.

Histological staining was used as a tool for tissue type discrimination within the distracted callus. The histological sections were transferred to Superfrost Plus slides and resin was removed prior to staining. Each section was stained with Von Kossa and counterstained with Toluidine blue, which respectively identifies mineralized tissues in black and cartilaginous tissues in purple (Figure 2). After staining was complete, the sections were dehydrated and mounted using a resinous mounting medium (Entellan^®^, Merk Corporation, Burlington, MA, USA). Photographs of the histological sections were taken using a microscope (BX-40, Olympus^®^, Tokyo, Japan) at 4x magnification. Once the section was completely acquired, we were able to assemble the photographs via Photoshop^®^ to obtain an overall view of the section.

### 2.3. Microindentation

Prior to microindentation characterization, the indentation sections were glued on a microscope slide and polished with a polishing machine ESC-200-GTL (ESCIL^®^, Chassieu, France) with carbide papers (P600, P1200, P2500). Subsequently, finer polishing was performed with the use of diamond slurry. A total of three particle sizes (3, 1, and 0.25 µm) were applied in succession. The samples were cleaned ultrasonically with distilled water after each polishing step. Microindentation was performed using a Tester NHT^2^ (Anton Paar^®^, Graz, Austria) with a Berkovich diamond indenter at a room temperature of 23 °C. The tip used has a diameter of 120 nm, an elastic modulus of 1141 GPa, and a Poisson’s ratio of 0.07. This instrumentation is mounted with a high-resolution microscope that allowed the positioning of the indenter tip precisely on the tissues of interest and avoided surface irregularities (e.g., porosity). Before each series of tests, a calibration test was made on a fused silica reference sample; the mean values obtained were 71.3 ± 1.6 GPa, the reference value being 72 GPa. A trapezoidal load function was used with a maximum load of 25 mN applied on the longitudinal direction and at a constant loading and unloading rate of 50 mN/min, which was separated by a 30 s holding phase. The 30 s holding period was selected to minimize the effect of creep and viscoelasticity [22], and the peak force was chosen to reduce the effects of surface roughness [23]. The hardness H and the elastic modulus E of the material were calculated using the Oliver and Pharr (OP) method [24]. In the OP method, the stiffness (S) at peak load was calculated as the slope of the unloading curve. In this study, the reduced modulus $E_{r}$ was determined by the region between 98% and 40% of the unloading portion of the curve at maximum load. The reduced modulus was then calculated according to the following equation:

(1) $$E_{r}=\frac{\sqrt{\pi }}{2\beta }\frac{S}{\sqrt{A_{c}}}$$

where S is the unloading stiffness, $A_{c}$ is the contact area, and β is a dimensionless correction factor that considers the non-axisymmetric of the tip. For a Berkovich type indent, it was evaluated at 1.034 [25].

The elastic modulus E of the specimen was then obtained from the following relation:

(2) $$\frac{1}{\mathrm{Er}}=\frac{\left(1-\nu_{s}^{2}\right)}{E}+\frac{\left(1-\nu_{i}^{2}\right)}{E_{i}}$$

where $E_{i}$ and $\nu_{i}$ are the known elastic modulus and Poisson’s ratio of the indenter and $E_{r}$ is the reduced modulus. In this study, the Poisson’s ratio was 0.3 for all mineralized tissues as it is the standard value for bone tissue [26].

The contact hardness is the peak load divided by the contact area, as described by the following equation:

(3) $$H=\frac{P_{\max}}{A_{c}}$$

where $P_{\max}$ is the maximum load and $A_{c}$ is the contact area.

#### 2.3.1. Regions of Interest (ROI)

**Figure 3:**
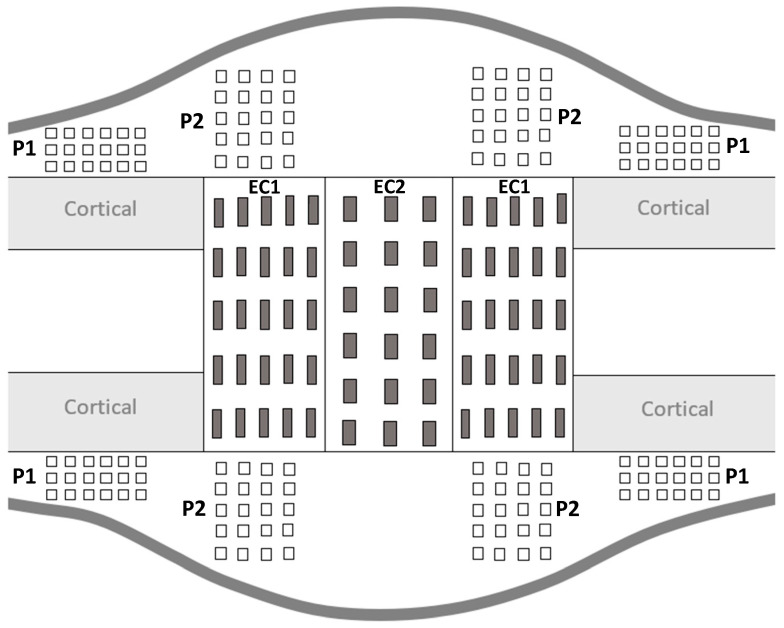
Schematic representation of distracted callus section in a longitudinal plane.

During the consolidation phase, sequential mineralization of osteoids occurs, starting at the host bone margins and advancing toward the center part where a fibrous island is present—called the fibrous interzone (FIZ) [2,27,28,29]. At the end of the distraction phase, five zones can already be discriminated in between the native cortical bone: a central FIZ; two primary mineralization fronts (PMF), which are on either side of the FIZ and contain a high density of proliferating osteoblasts; and two intermediate zones containing microcolumn formation (MCF) [28,29,30]. As the consolidation process continues, the FIZ is progressively reduced until complete disappearance [4,31]. This means that the FIZ is the last part to undergo mineralization, so the bone tissue present in this area is the youngest [27]. Moreover, both periosteum and endosteum contribute to the mineralization process, with a more important contribution from the periosteum [31]. Therefore, to mechanically characterize the heterogeneity of bone-forming tissue, several ROI were defined for the indentation matrices (Figure 3).

For all the ROI, the indentation matrices were made on the proximal and distal sides as well as on the medial and lateral sides of the distracted callus. The distracted callus was delimited in two parts, leading to the characterization of the outer callus (periosteal callus) and the internal callus (endosteal callus). The periosteal callus was composed of two ROI called P1 and P2. The P1 ROI corresponded to the area adjacent to the native cortical bone. A 3 × 6 indentation matrix (600 µm × 1500 µm) was used to characterize this region. The P2 ROI represented the osteotomized area in the periosteal callus and a matrix of 5 × 4 indentations (1200 µm × 900 µm) was applied to describe it. The endosteal callus was also subdivided into two ROIs named EC1 and EC2. The EC1 ROI represented the MCF zone and a matrix of 5 × 5 indentations (1600 µm × 2000 µm) was used. The EC2 region corresponded to the central part of the distracted callus (FIZ) and was described by a matrix of 6 × 3 indentations (2500 µm × 1200 µm). Furthermore, on each visible native cortices, a matrix of 3 × 6 indentations (280 µm × 325 µm) was also made. P1 and P2 refer to the periosteal callus and EC1 and EC2 define the endosteal callus.

#### 2.3.2. Tissue Type

We focus our interest on mineralized tissue present within the distracted callus. Interestingly, throughout DO, both endochondral and intramembranous bone formation took place [32,33]. Endochondral ossification occurs through the deposition of a transient cartilage tissue, leading to the formation of a calcified cartilage structure. This type of ossification is seen during the distraction and consolidation phases [2,6]. It has been reported that calcified cartilage is present in between the FIZ and the newly-mineralized membranous bone [6,34,35]. In this study, after observation of the histological staining, we realized that the calcified cartilage areas were randomly arranged and represented a small mineralized zone. Given this, no specific ROI were chosen for this tissue type characterization.

Moreover, intramembranous ossification is characterized by the direct differentiation of mesenchymal stem cells into osteoblasts, which will lead to the synthesis of woven bone tissue and, later, trabecular bone [36,37,38]. During DO, intramembranous ossification remains the most predominant mechanism [1,2,34] and we were able to observe bone-forming tissue inside all the ROIs previously described (periosteal and endosteal callus). In this study, the term, trabecular bone, included the mechanical characterization of both woven bone (immature) and lamellar bone (mature bone).

We also characterized cortical bone tissue, which is a highly-organized mineralized tissue. This was observed in the osteotomized native cortices ROI and was used for references values. The data obtained for trabecular bone were compared with the mean value of cortical bone evaluated from all indentations.

### 2.4. Data Processing and Statistical Analysis

The elastic modulus (E) and hardness (H) of the mineralized tissue as a function of the consolidation time allowed us to establish the evolution profile of the mechanical properties over time. For cortical and trabecular bone, characterization was possible at each time point. However, for calcified cartilage, a total of 40 points per section was recorded only at 31-DPS and 45-DPS with the help of histological staining. In the 59-DPS distracted calluses, the calcified cartilage surface was scattered or, when present, was not large enough to allow indentation.

In addition, the analysis of mechanical properties as a function of the ROI was determined to analyze the spatial variations within the distracted callus. This analysis was done solely on trabecular bone. It is important to note that the number of points per indentation matrix for trabecular bone characterization was dependent on bone callus geometries and observation time. For instance, within the EC2 ROI, fewer points were obtained at 31-DPS than at 59-DPS, as the bone regenerating process during DO takes place centripetally [14,27,28,31]. Moreover, for all ROIs except the EC2 area, we calculated the mean value by gathering measurements from all corresponding sub-regions. As an example, for P2, data from the four sub-regions have been averaged.

The resin used for embedding was mechanically characterized and obtained a mean value of 3.9 ± 0.13 GPa. We decided to set aside all values with an elastic modulus lower than 5 GPa to be sure to characterize mineralized tissue and not something else.

The data obtained for the quantification of mechanical parameters were expressed as mean ± standard deviation (SD) and whisker plots were used to display the various results. Normality tests were performed on the data with a Shapiro–Wilk test (*p* > 0.05). Since normal distribution was not respected, we used a non-parametric Kruskal–Wallis test to see differences between regions. If the latter obtained a *p*-value less than 0.05, multiple pairwise comparisons were performed using Dunn’s test with Bonferroni correction. For the calcified cartilage dataset, a Mann–Whitney U test was applied. Differences were considered significant at a *p*-value of 0.05 regardless of the statistical test applied. All statistical analyses were performed using XLSTAT software.

## 3. Results

### 3.1. Temporal Variation of Mineralized Tissue within the Distracted Callus

The elastic modulus (E) and hardness (H) mean values of cortical bone tissue, calcified cartilage, and trabecular bone as a function of the time of consolidation were measured at 31-DPS and 45-DPS. At 59-DPS, only cortical tissue and trabecular bone were measured.

**Figure 4:**
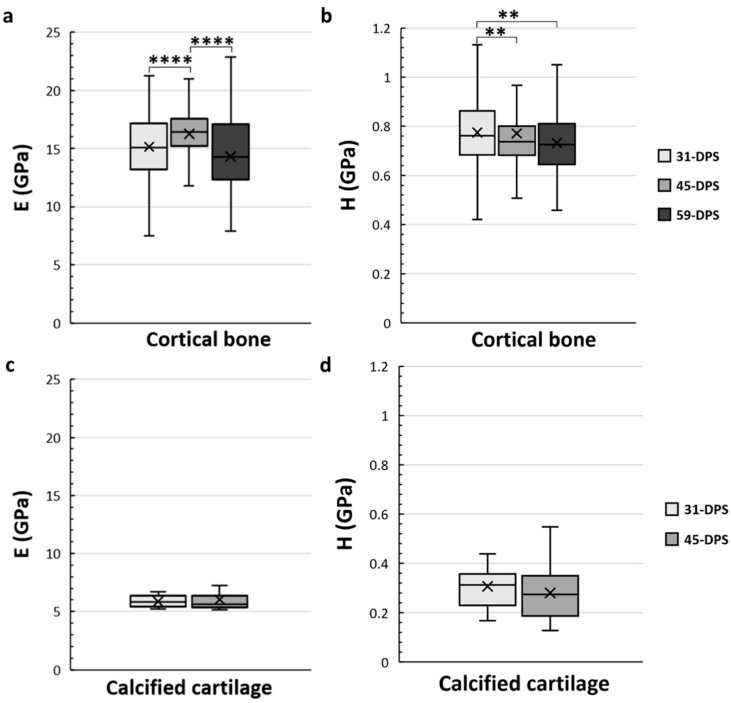
Temporal variations of elastic modulus (E, in GPa) (**a**,**c**) and hardness (H, in GPa) (**b**,**d**) of the native cortical bone (**a**,**b**) and calcified cartilage (**c**,**d**) inside the distracted bone. (**a**) At 45-DPS, a statistical difference for E is seen compared with 31-DPS and 59-DPS. (**b**) The data for H show a statistical difference at 31-DPS compared with the 45- and 59-DPS. (**c**,**d**) For E or H parameters, the data did not show any differences over time. x = mean value, **** *p* < 0.0001 and ** = *p* < 0.01.

The cortical bone showed significant time-dependent differences in E and H (Figure 4a,b). The mean values of E were 15.2 GPa (±3), 16.2 GPa (±2), and 14.3 GPa (±3) at 31-DPS, 45-DPS, and 59-DPS respectively. The mean value of E obtained at 45-DPS was significantly higher compared with the other time-points (*p* < 0.0001). Concerning H data, the mean values were 0.775 GPa (±0.14) at 31-DPS, 0.771 GPa (±0.27) at 45-DPS, and 0.732 (±0.12) at 59-DPS. H showed a significant difference at 31-DPS compared with 45-DPS (*p* = 0.006) and 59-DPS (*p* = 0.003). For the calcified cartilage, mechanical properties (E and H) were stable over time. The mean values for E were 5.9 GPa (±0.5) at 31-DPS and 6 (±1.1) at 45-DPS (Figure 4c). For H, the mean values were 0.307 (±0.08) and 0.280 (±0.11) at 31-DPS and 45-DPS, respectively (Figure 4d).

**Figure 5:**
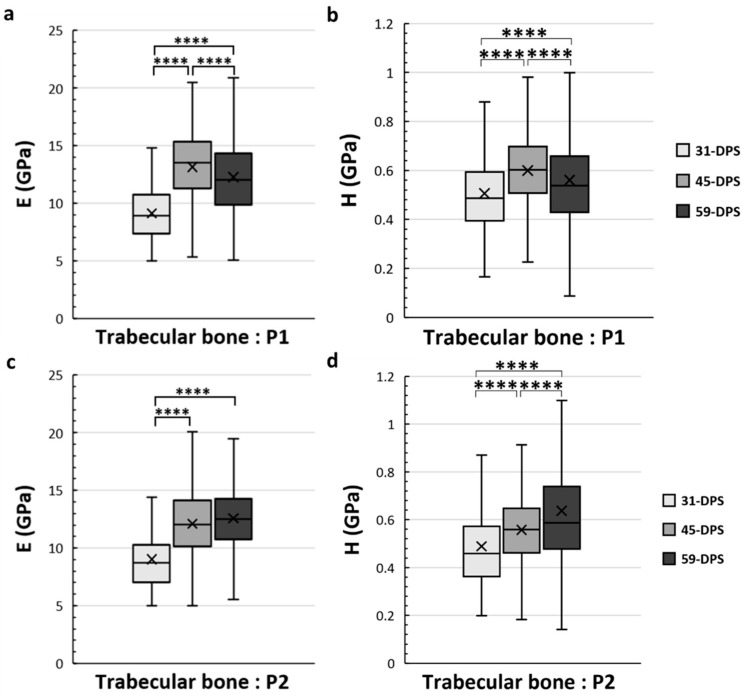
Elastic modulus (E, in GPa) (**a**,**c**) and hardness (H, in GPa) (**b**,**d**) in the periosteal callus (P1 and P2 ROIs) for trabecular bone. In the P1 region, E (**a**) and H (**b**) showed a significant increase over time with a significant peak at 45-DPS. In the P2 region, at 31-DPS, the E showed a significant difference compared with 45-DPS and 59-DPS (**c**). Regarding the H mean values, a significant increase was seen throughout the time of consolidation. x = mean value, **** *p* < 0.0001.

Concerning the trabecular bone tissue, for both E and H the values were assessed in the different ROIs that we have described above in the methodology section. In the periosteal callus, we observed a similar evolution profile for the mechanical properties of E and H in the P1 ROI (Figure 5a,b). The mean values of E increased significantly from 9.2 GPa (±2) to 12.3 GPa (±4) (*p* < 0.0001) during the consolidation period between 31-DPS and 59-DPS. Regarding H, the same observation could be seen since the average values were 0.506 GPa (±0.17) and 0.560 GPa (±0.22) at 31-DPS and 59-DPS, respectively (*p* < 0.0001). Surprisingly, at 45-DPS, a peak was reached for E (13.2 GPa (±3)) and H (0.600 GPa (±0.15)), which were significantly higher compared with the other time-points (*p* < 0.0001). In the P2 ROI, a significant increase of E was observed between 31-DPS and the two other time-points (*p* < 0.0001). No difference was observed for E between 45-DPS and 59-DPS (Figure 5c). The mean values of E during consolidation time-points were 9 GPa (±3), 12.1 GPa (±3), and 12.6 GPa (±3) at 31-DPS, 45-DPS, and 59-DPS, respectively. For H, we notice a significant increase over time (*p* < 0.0001) (Figure 5d). The mean values rose significantly from 0.489 GPa (±0.18) to 0.557 GPa (±0.16) at 31-DPS and 45-DPS, respectively (*p* < 0.0001). At 59-DPS, the mean value obtained was 0.637 GPa (±0.37) and was significantly higher than those at 31-DPS (*p* < 0.0001) and 45-DPS (*p* = 0.040).

**Figure 6:**
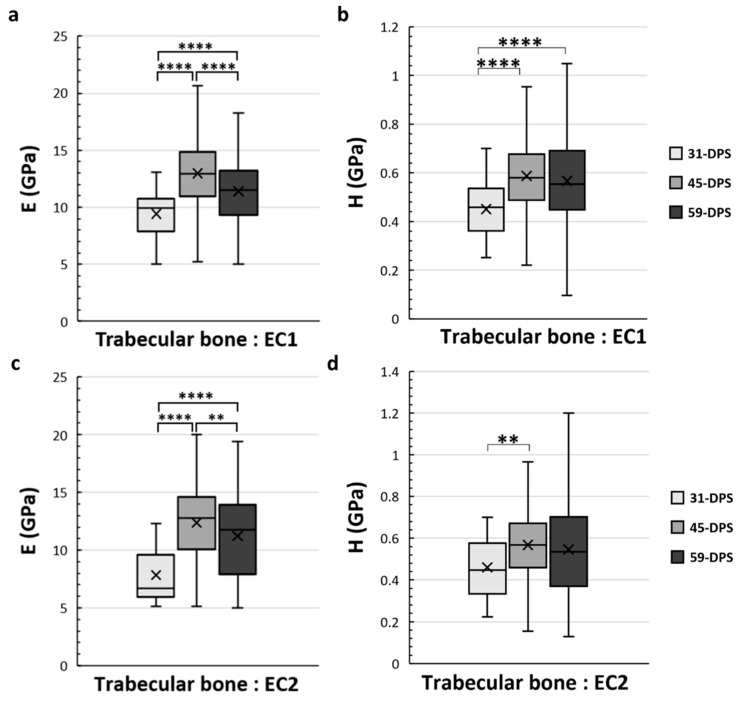
Elastic modulus (E, in GPa) (**a**,**c**) and hardness (H, in GPa) (**b**,**d**) in the endosteal callus (EC1 and EC2 ROIs) for trabecular bone. (**a**) In the EC1 region, E showed a significant increase over time with a significant peak at 45-DPS. (**b**) For H parameters in EC1 ROI, statistical differences were seen between 31-DPS and the two other time-points. (**c**) In the EC2 region, at 31-DPS, E showed a significant difference compared with 45-DPS and 59-DPS. (**d**) For the H parameter, the only statistical difference was observed between 31-DPS and 45-DPS. x = mean value, ** *p* < 0.01 and **** *p* < 0.0001.

In the EC1 and EC2 ROIs, which correspond to the endosteal callus, the mechanical properties of the trabecular bone also evolve over time (Figure 6). In the EC1 ROI, the evolution profile for E was similar to the P1 ROI. The data acquired at 31-DPS (9.4 GPa (±2)) were significantly lower than the data obtained at 45-DPS (13 GPa ± 3, *p* < 0.0001) and 59-DPS (11.4 GPa (±3), *p* < 0.0001). However, we also noticed a significant peak at 45-DPS compared with the mean value obtained at 59-DPS (*p* < 0.0001). For H, the mean value at 31-DPS (0.451 GPa (±0.11)) was significantly lower from those at 45-DPS (0.587 GPa (± 0.16), *p* < 0.0001) and 59-DPS (0.567 GPa (±0.2), *p* < 0.0001), but no difference was seen between 45-DPS and 59-DPS. In the EC2 ROI, the evolution of E was also similar to P1 and EC1 ROIs (Figure 6c). At 31-DPS, the mean value for E was 7.8 GPa (±2) and was significantly lower compared with the other times (*p* < 0.0001). We also observed a significant increase in the mean value at 45-DPS (12.4 GPa (±3)), followed by a significant decease at 59-DPS (*p* = 0.008). For the H, the only difference seen was between 31-DPS (0.461 GPa (±0.15)) and 45-DPS (0.568 GPa (±0.17)) (*p* = 0.007) (Figure 6d).

### 3.2. Spatial Variation of Mineralized Tissue within the Distracted Callus

**Figure 7:**
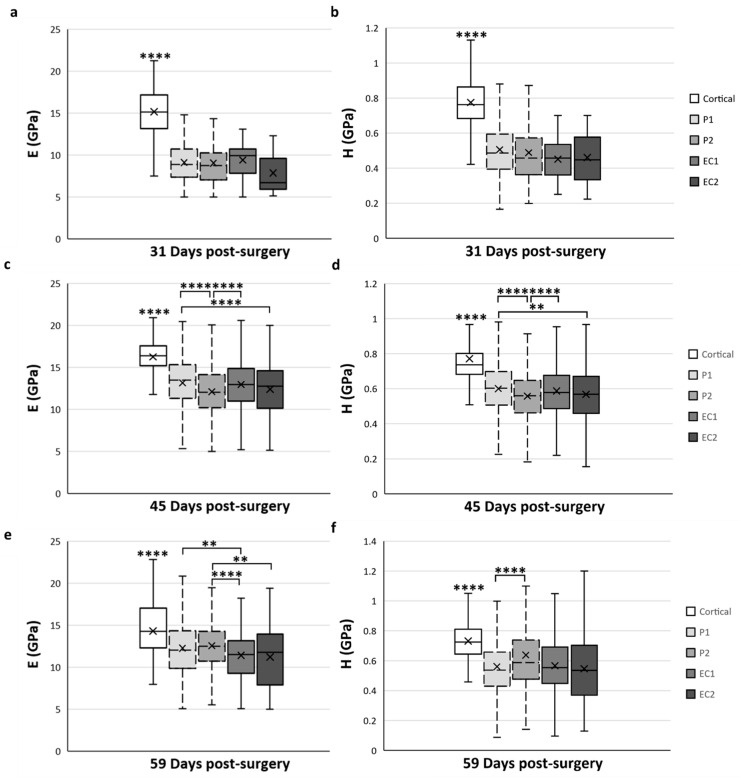
Spatial variation of elastic modulus (E) (**a**,**c**,**e**) and hardness (H) (**b**,**d**,**f**) of trabecular bone tissue depending on the ROI located in the distracted callus. (**a**,**b**) Mean values obtained for E (**a**) and H (**b**) at 31-DPS. No statistical differences are found between the periosteal callus (P1 and P2 ROI) and endosteal callus (EC1 and EC2 ROI). (**c**,**d**) E and H showed the same spatial variation profile. The most central ROI (EC2 and P2) showed significant differences compared with the ROI close to the native cortices (P1 and EC1). (**e**,**f**) At 59-DPS, differences are found between E and H for spatial variation. For H values, the P2 area was statistically higher than P1. For E, the periosteal callus (P1, P2) showed significantly higher data than the endosteal callus. x = mean value, ** *p* < 0.01 and **** *p* < 0.0001.

As the mechanical properties of the calcified cartilage were not measured in a specific ROI and did not change over time, we only focused on the cortical bone and trabecular bone tissue types for the spatial variation analysis (Figure 7). The mean data obtained for cortical bone in the native cortical ROI were always significantly higher compared with all ROI and at any time-point of the analyses (*p* < 0.0001) (Figure 7).

At 31-DPS, no significant differences were noticeable for the E and H parameters among all ROIs (Figure 7a,b). The mean values of E ranged from 7–9 GPa with a minimal value of 7.8 GPa (±3) in the EC2. For H, the mean values in the P1, P2, EC1, and EC2 ROIs were also homogeneous and ranged between 0.451 and 0.506 GPa.

Interestingly, the E and H parameters of trabecular bone at 45-DPS show the same profile in ROI differences. The mean value for the P1 ROI was significantly higher compared with P2 ROI (*p* < 0.0001) and EC2 ROI (*p* < 0.001 for H and *p* < 0.0001 for E) (Figure 7c,d). In addition, the mean values of E and H were significantly higher in the EC1 ROI than in the P2 region (*p* < 0.0001). The P1 and EC1 ROIs showed a mean value around 13 GPa for E and a mean value around 0.600 GPa for H, while in the P2 and EC2 ROIs, the mean values were around 12 GPa and 0.560 GPa for E and H, respectively.

After 59-DPS, no differences in E were observed within the two ROIs of the periosteal callus (P1 and P2). The same trends could be observed in the ROIs of the endosteal callus as no differences were notable between the EC1 and EC2 ROIs. However, a significant difference was found between the periosteal and endosteal calluses (Figure 7e). In the P1 ROI, the mean value was significantly higher compared with the EC1 ROI (*p* = 0.002) and, for the P2 ROI, we observed a significant rise in the E mean value compared with the EC1 (*p* < 0.0001) and EC2 (*p* = 0.003) ROIs.

Regarding H, the only significant difference was observed within the periosteal callus between the P1 and P2 ROIs (Figure 7f), where P2 ROI obtained a significantly higher value than P1 (*p* < 0.0001).

## 4. Discussion

The elastic modulus and hardness at the microscale of the mineralized tissues in the distracted callus are poorly understood. Interestingly, it has been shown that these intrinsic parameters are a key factor in the mechanical response of the structure at a macroscale [39,40]. The aim of this study was therefore to characterize the elastic modulus and hardness of the calcified cartilage and the trabecular and the cortical bone present in the distracted callus following bone lengthening. We used a microindentation assay to obtain the mechanical properties at the microscale level of the periosteal (outer) and endosteal (internal) callus. Moreover, the distracted callus is composed of several mineralized tissues at different stages of formation, so we used histological staining to locate the tissue of interest inside the distracted callus. This methodology allowed us to recognize and characterize trabecular bone from calcified cartilage during the bone regenerating process in specific areas within the distracted callus. In this study, for the first time, we simultaneously characterized the endosteal and periosteal calluses to describe the temporal and spatial variations of the bone tissue during the consolidation process in the DO model.

### 4.1. Temporal Variation of the Mineralized Tissues

The first aspect analyzed in this experiment was the evolution of the mechanical properties of the mineralized tissue within each ROI in a time-dependent manner. Before exploring this aspect, we need to present the relationship between the mechanical parameters (elastic modulus and hardness) that are intrinsic to the composition of the bone extracellular matrix (ECM). Elastic modulus can be linked to the interconnectivity of the organic and inorganic parts of the ECM. Whereas, for hardness, even though it is not completely confirmed, it seems that it is mostly connected to the inorganic part of the ECM represented by the mineral composition [41]. Given this, by examining these mechanical parameters, we were able to quantify how bone regeneration had progressed throughout the consolidation phase of DO.

Now that these relationships are established, we can focus on the evolution profile over time of the elastic modulus and hardness within the distracted callus. Three tissues of interest were analyzed during this study: native cortical bone, trabecular bone, and calcified cartilage. We started by looking at the evolution profile of the cortical bone of the native cortices close to the osteotomized area. The mean values obtained for elastic modulus were comprised between 14 and 16.5 GPa for elastic modulus and between 0.732 and 0.775 GPa for hardness values. These values agreed with the studies in the literature that use a microindentation assay on rat-embedded femurs [18]. Interestingly, even at the level of native cortical bone, we could observe a significant variation over time for both parameters. These observations were not surprising as it has been shown that DO also affects the pre-existing bone. Indeed, some studies have shown the reactivation of the bone formation process in the native cortices by enhancing osteogenic activity, leading to increased osteoid volume [4,30,42].

As expected, for both elastic modulus and hardness, the mechanical properties inside the distracted callus tend to increase over time regardless of the ROI. However, for calcified cartilage, no variation between 31-DPS and 45-DPS was seen for either of the mechanical parameters. Surprisingly, in the literature, no study has yet characterized the calcified cartilage during bone formation regardless of the regenerating process. Therefore, comparisons were directed toward calcified cartilage present in articular cartilage tissue in the rat model. For elastic modulus, measurements following the frontal plane oscillate between 3 and 6 GPa, which were in the same range as our values [43]. Moreover, we could observe that calcified cartilage presented lower and more stable values over time for elastic modulus compared with the other mineralized tissues. This can be explained by a distinct ECM organization compared with bone tissue [39], thereby leading to lower interconnectivity between the collagen fibrils and mineral content [44]. Concerning hardness values, the same observation over time could be made and, when compared with other studies on bone regeneration processes, no information was found in the literature. However, it is known that the mineral composition of the crystals remains similar to the mineral part of the bone tissue [44,45]. Given this, we could suppose that the lower values obtained for hardness in the calcified cartilage could be due to the nature of this tissue: as the mineral phases remain the same between bone and calcified cartilage, the mineral content varies [45]. Indeed, throughout the bone regeneration process, this tissue type is transient, meaning that it is not created to stay and grow for a long time period and, during endochondral ossification, it is known that calcified cartilage secreted by hypertrophic chondrocytes is degraded rapidly by the osteoclast [46] and serves as a support for the formation of bone trabeculae [47]. This could explain the constant nature of the values obtained over time for both elastic modulus and hardness.

The second tissue of interest characterized inside the distracted callus was the trabecular bone. We noted that the literature on the mechanical properties during a bone regenerative process remains limited. To our knowledge, such analyses have been carried out in only two studies. One was done by using a bone fracture model and focused on periosteal callus [17], and the other used a bone transport model to characterize the distracted callus and docking-site [18].

In this study, when we gathered all the elastic modulus values obtained for trabecular bone regardless of the ROI selected, we observed that values ranged from 6–13 GPa. These values were similar to the ranging observed in the literature, which ran from 6–18 GPa and from 4–18 GPa for Manjubala et al.’s and Mora-Macías et al.’s studies, respectively. For hardness, our values ranged from 0.064–4.589 GPa. No comparison could be made on that range with the literature, but the average values and standard deviation obtained in our study were similar to those in the study by Manjubala and al. We also looked at the coefficient of variation (COV) of the values, which represents the dispersion around the mean. This allowed us to highlight the heterogeneity of the mechanical properties of bone formation and, indirectly, the consolidation process. Mineralization is governed by a phenomenon called heterogeneous nucleation, which includes a mechanism of primary nucleation—where the mineral deposit is initiated via the transformation of a liquid solution (containing a high concentration of calcium and phosphate ions) into a solid phase—and a mechanism of secondary nucleation, which uses these first mineral deposits as nucleation sites for the formation of other apatite crystals [47]. Our data obtained a COV between 20–31% for elastic modulus and between 24.5–58% for hardness. When comparing with the literature, we observed that our COV is less spread, for both mechanical parameters, which could be explained first by the bone regeneration model studied and secondly by the shorter time period of analyses.

**Figure A1:**
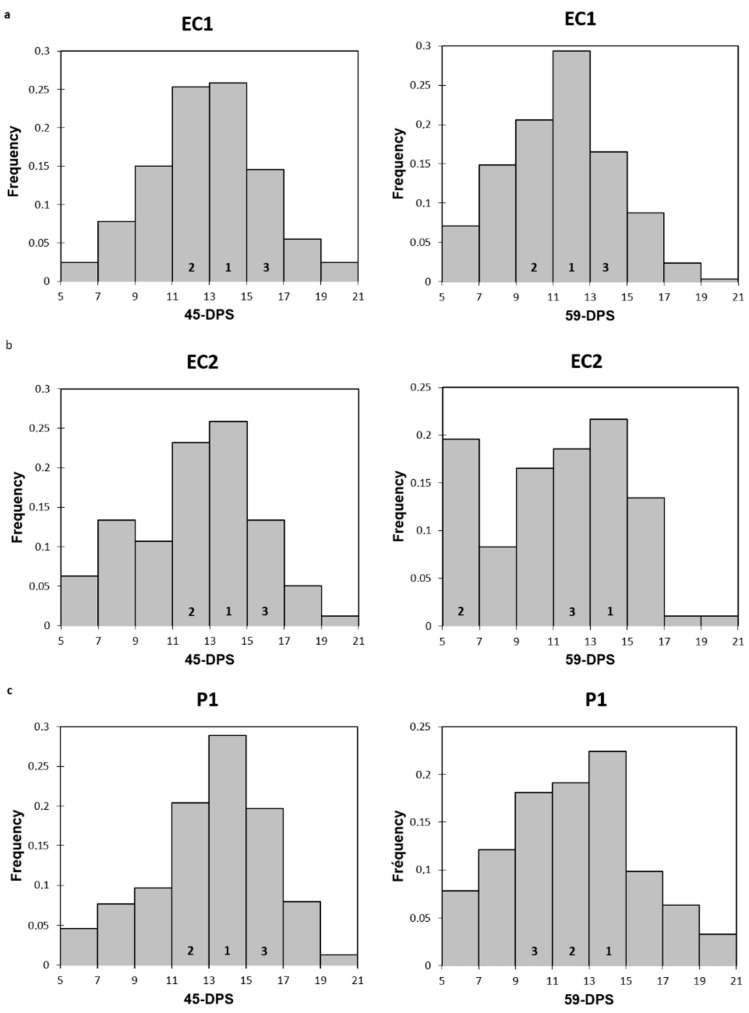
Histograms representing the frequency distribution of elastic modulus at 45 and 59-DPS for EC1 (**a**), EC2 (**b**) and P1 (**c**). Numbers 1, 2 and 3 represent the top three ranking.

When we now look at the evolution of the trabecular mechanical properties within each ROI in a time-dependent manner, we could observe that, regardless of the ROI and for both mechanical parameters analyzed, the values increased over time and were higher at 59-DPS compared with 31-DPS. These observations were in accordance with the consolidation phase of DO, which tends to increase the bone volume tissue within the distracted callus until complete bridging by creating a dense network of trabecular bone. Interestingly, for the endosteal callus (EC1 and EC2) and the P1 ROI of the periosteal callus, the same evolution profile was apparent for the elastic modulus; the values peaked at 45-DPS and decreased at 59-DPS. We may suppose that these significant differences are due to the extent of apposition and mineralization of bone-forming tissue between these two time-points. During bone lengthening, mineralization occurs in a centripetal way, meaning that it starts close to the native cortices and progresses toward the center of the callus [27]. Moreover, this could support the idea that more newly-formed woven bone was deposited at 59-DPS, but this was not yet completely remodeled, leading to less mechanically rigid areas compared with 45-DPS. These observations are in accordance with consolidation during the bone lengthening process, where gradual remodeling of the woven bone over time is seen, resulting in the formation of a mature, lamellar bone [2,3,30,42,48]. To reinforce this hypothesis, we looked at the frequency distribution of the data within each ROI of the endosteal callus (EC1 and EC2) and P1 ROI at 45- and 59-DPS (summarized in Appendix A Figure A1). At 45-DPS, all ROIs showed a similar distribution profile over the top three range, the highest being the one with the values between 13 and 15 GPa. However, at 59-DPS the histograms frequency changed for all the ROIs. For P1, the first two ranges did not change compared with 45-DPS, but the third range went from 15–17 GPa to 9–11 GPa. For EC2, the second range changed to the 5–7 GPa range. Concerning EC1, this idea was strengthened as it was the first range that changed to the 11–13 GPa range.

Regarding the hardness evolution profile during the consolidation phase of DO, the values obtained within the periosteal and endosteal callus did not show a particular pattern, as was the case for elastic modulus. In the endosteal callus, for both ROIs, a quite similar evolution profile was observed as the hardness values increased significantly compared with the 31-DPS time-point, but no significant differences were found between the 45- and 59-DPS time-points. In the periosteal callus, the P1 ROI, which was closer to the native cortices, showed the same evolution profile as for elastic modulus, with a significant peak at 45-DPS and a decrease at 59-DPS. Interestingly, for the P2 ROI, a significant and continuous increase over time was observed. We can assume that this increase over time was established so as to mechanically stabilize the distracted callus. Experimental studies have shown that the mechanical environment detected by the distracted callus is an important factor in the bone-healing process [7,49,50] and that shear stress could disturb or inhibit osteogenesis and lead to fibro-cartilage or fibrous tissue [50]. Cleas et al. [51] showed that during the consolidation phase of DO, shear movement reduced bone formation and delayed bone healing. They supposed that the disruption of vascularization might cause that observation by reducing the density of blood vessels present in the distracted callus in the consolidation phase. Given this, we could suppose that the strategy of the bone regeneration process during the consolidation phase of DO was to increase the hardness of the outer callus and to minimize or reduce the shear stress of the distracted callus.

In the end, this temporal analysis of bone-forming tissue reinforced the idea that the consolidation process during DO is not continuous or gradual and leads to heterogeneity in the mechanical properties of the distracted callus.

### 4.2. Spatial Variation of the Mineralized Tissues

The second aspect analyzed in this experiment was the spatial variations of the mechanical properties of the distracted callus over time in a ROI-dependent manner. For this part of the experiment, we focused our interest on only the cortical bone and the trabecular bone, since there were no no specific ROI selected for calcified tissue. The cortical bone was measured in the native cortices close to the osteotomized area and the trabecular bone was estimated within four distinct ROIs, leading to the mechanical characterization of the periosteal callus (P1 and P2) and the endosteal callus (EC1 and EC2) at the same time.

In our study, we could observe that for both mechanical parameters of the cortical bone, the values were significantly higher compared with the other tissues present within the distracted callus, regardless of the ROI selected and at any time-point of the experiment. This finding was not surprising since complete consolidation (mineralization and bridging) is a long process [48], and even more time is needed for the distracted callus to undergo a functional remodeling process to form a normal cortico-medullary architecture that resembles native cortical bone [3,52]. Furthermore, a similar finding was reported by Mora-Macías et al., who found that the elastic modulus obtained for trabecular bone formed within the callus at day 504 of the consolidation phase after bone transport process was only 75% of the value of the native cortices [18].

Now, when we focused our interest on the mechanical properties of the trabecular bone, we could see some variation between the outer (periosteal) and the internal (endosteal) callus. At 31-DPS, regardless of the ROI, we observed no statistical differences for both mechanical parameters (elastic modulus and hardness). This highlights the consistency of the consolidation process by showing that the periosteal and endosteal callus were in similar states of consolidation. Indeed, the mean values were homogeneous inside the distracted callus and ranged between 7.8 and 9.5 GPa for elastic modulus and between 0.451 and 0.506 GPa for hardness. However, it is important to note that the lowest values were apparent in the most central region of the endosteal callus (i.e., EC2). This observation was in accordance with the literature, as this ROI corresponds to the FIZ of the distracted callus where high mechanical stress induced cells to stay in a proliferative state, and which is the last area to be colonized by trabecular bone [29].

Regarding the spatial differences at 45-DPS, we could observe the centripetal formation just by looking at the mechanical properties of the bone-forming tissue. In this study, the ROI close to the native cortices (P1 and EC1) showed higher mechanical properties compared with the rest of the zones, and similar spatial variation profiles were obtained for elastic modulus and hardness. For the periosteal callus, the P1 ROI was different from the P2 ROI and EC2 ROI, which corresponded to the most central part of the callus. We also found a significant difference between the EC1 ROI and P2 ROI. These results highlight the natural bone formation process observed during the consolidation phase in DO. As discussed earlier, it has been shown that mineralization starts in areas close to the native cortices and goes through the center of the distracted callus [5,53,54,55]. Thus, our results showed that the periosteal and endosteal callus close to the native cortices were at a more advanced stage than the rest of the callus.

Finally, at 59-DPS, different profiles were observed for elastic modulus and hardness parameters. For elastic modulus, the values measured in the periosteal callus increased significantly compared with those in the endosteal callus. We could link these observations with descriptions of the bone regenerative process in the literature. The outer edge of the distracted callus tends to form tissue with cortical-like bone properties during the consolidation phase of DO [56]. Moreover, if we looked at the hardness, the only differences seen at 59-DPS were inside ROIs of the periosteal callus. We noticed a sharper increase for the most central area (i.e., P2). We could suppose that the strategy implemented by the process is to quickly stabilize the newly-formed callus, since the latter is a mechanically overstressed zone [49,57]. Consequently, the bone regenerative process tends to reinforce the external callus to create and maintain a permanent low-strain environment in the distracted callus.

In the end, the key message is that by measuring the mechanical properties in different ROIs in the callus as it underwent successive stages of consolidation, we were better able to understand how the consolidation process was initiated. Our finding supports the previous existing idea that consolidation during DO begins by first mineralizing the “microcolumn formation zone” before mineralization of the central area. Furthermore, from what we observed, the regenerative strategy happens in two successive stages. The first stage is to increase the rigidity and hardness of the zones close to the native cortices. The second stage is to increase the rigidity and hardness of the outer edge of the distracted callus. Then, the strategy aims to gradually increase the overall mechanical properties of the tissues located in the core of the callus.

All the results gathered in this study enrich the understanding of the regeneration process from a mechanical point of view. Nevertheless, there are still some limitations concerning the mathematical model we chose. The model used was developed by Oliver and Pharr and suggests that the tissues present within the regenerate have a purely elastic behavior. However, mineralized tissues can express viscoelastic behavior. The Oliver and Pharr model was chosen because it is the one most used in the literature, which facilitates comparisons. In addition, it has been shown that the viscoelastic effects of tissues are less important in hard mineralized tissues than soft tissues [18]. It could be interesting to explore the viscoelastic properties of trabecular bone and calcified cartilage during DO to characterize the elastic, plastic, and viscous contribution of bone material [58]. Moreover, the number of animals could be greater and an evaluation of later periods of the consolidation process could be useful.

## 5. Conclusions

In conclusion, this study is a first innovative approach since it is the first time that both temporal and spatial variations of the mechanical properties within the distracted callus have been characterized during DO. We have shown that the mechanical properties of the trabecular bone within the periosteal and endosteal callus increased with time, and that the bone regeneration process includes areas with different mechanical properties, thereby highlighting the variations in mechanical parameters from a spatial perspective. Furthermore, this study allowed us to characterize the consolidation process from a purely mechanical point of view. These observations could be connected to the evolution profiles observed in the literature from a biological point of view. Moreover, all these data are an important source of information for the numerical modeling of the regeneration process, as we have shown that there is heterogeneity of trabecular bone-forming tissue at all points internally, temporally, and spatially. The evolution profile of the mechanical parameters over time suggests that the consolidation process does not involve a continuous mineralization, but rather a coordination between apposition, mineralization, and remodeling of the tissue during the consolidation phase. We also observed that the process starts by reinforcing the external callus to reduce the general mechanical strain of the callus. It would therefore be judicious to implement such information in a numerical model. An improvement of the models would help researchers to optimize the animal pre-test phase and thus to participate in the reduction of the number of animals used in pre-clinical studies. Finally, these data are necessary to understand the regeneration process. By accurately characterizing the different tissues, a more precise reflection on the improvement of the bone regeneration process of the DO could be conducted.

## Data Availability

The data presented in this study are available on request from the corresponding author.
